# Evaluation Report of the Colistin Broth Disk Elution Method with *Acinetobacter baumannii* Isolates from a Low-Resource Setting

**DOI:** 10.1128/spectrum.00871-22

**Published:** 2022-08-29

**Authors:** Swati Sharma, Tuhina Banerjee, Rahul Garg, Padma Das

**Affiliations:** a Department of Microbiology, Institute of Medical Sciences, Banaras Hindu University, Varanasi, Uttar Pradesh, India; b Department of Microbiology, All India Institute of Medical Sciences, Raipur, Chattisgarh, India; Instituto Oswaldo Cruz

**Keywords:** CBDE, BMD, *A. baumannii*, agreement

## Abstract

The rapid emergence of drug resistance in Acinetobacter baumannii has put forward the use of colistin as a last-resort treatment for infections with A. baumannii. Empirical colistin use without prior susceptibility testing has been one of the factors that has been promoting drug resistance in low-resource settings. In this regard, while the advocated broth microdilution (BMD) method for colistin susceptibility testing is often considered cumbersome, the preferable colistin broth disk elution (CBDE) method has not yet been approved for A. baumannii. To prevent the underreporting of colistin susceptibility, we tested the CBDE method for A. baumannii and compared the results with those of BMD. A total of 125 A. baumannii, including 100 susceptible and 25 resistant isolates were tested via the CBDE method and compared with the standard BMD method. The essential agreement, categorical agreement, sensitivity, and specificity for CBDE were 97.6% (*n* = 122), 98.4% (*n* = 123), 100%, and 98.40%, respectively. The percentage of major error found was 1.6% (*n* = 2), and no very major error was found. CBDE in A. baumannii could be considered in low-resource settings.

**IMPORTANCE** The relatively cumbersome broth microdilution (BMD) method for routine colistin susceptibility testing has not been adopted, especially in low-resource settings, often leading to the underreporting of colistin susceptibility and the promotion of the empirical use of colistin. In this regard, the much-preferred colistin broth disk elution (CBDE) method has not yet been approved for A. baumannii. We evaluated colistin susceptibility via the CBDE method, compared the results with those of the BMD method in 125 A. baumannii isolates with various profiles, and inferred that the CBDE method using 50 μL inoculum could be helpful, at least in resource-limited setups, versus not reporting susceptibility testing for colistin.

## INTRODUCTION

Acinetobacter baumannii (A. baumannii) has emerged as a challenging global pathogen. The endemic status of this pathogen and the ever-increasing burden of carbapenem resistant A. baumannii (CRAB) have narrowed down therapeutic options to colistin in many critical cases. The approved method for the determination of colistin susceptibility for A. baumannii by the Clinical and Laboratory Standard Institute (CLSI) and The European Committee on Antimicrobial Susceptibility Testing (EUCAST) is the broth microdilution (BMD) method, which is labor-intensive and technically demanding. The cumbersome nature of this approach has led to the underreporting of colistin susceptibility in resource-poor, endemic setups, thus encouraging empirical use (1). In this context, the colistin broth disk elution (CBDE) method, which is easier to perform, has been approved for many multidrug resistant organisms (e.g., Enterobacterales, Pseudomonas aeruginosa) but not for A. baumannii (2). The 2,000-bed tertiary care hospital that participated in this study has already been challenged by outbreaks and the endemicity of CRAB in its intensive care units against a background of considerable empirical colistin use (3, 4). During the conduct of the study, analyses of colistin susceptibility against A. baumannii were not performed routinely by the diagnostic laboratory. Consequently, we tested the CBDE method for A. baumannii and compared the results with those obtained via the BMD method.

## RESULTS

**FIG 1:**
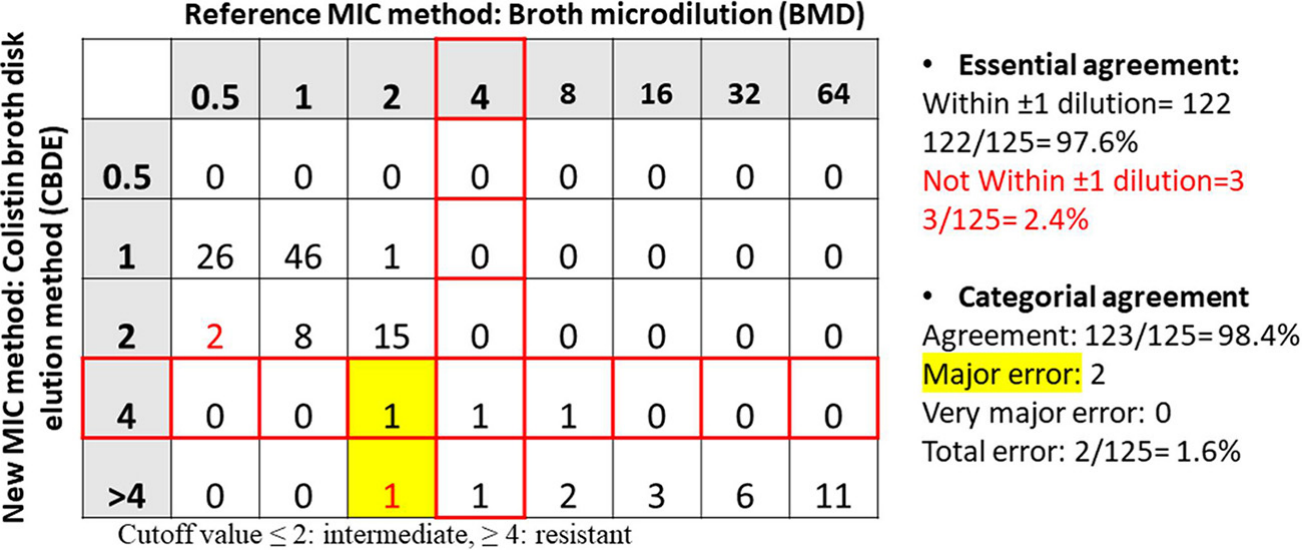
Scatterplot showing essential and categorical agreement between two test methods in A. baumannii isolates.

Among the 100 susceptible A. baumannii isolates, only 2 isolates were indicated as susceptible by the BMD method (minimum inhibitory concentration [MIC]: 2 μg/mL) but showed resistance with the CBDE method (MICs: 4 μg/mL and >4 μg/mL, respectively). The remaining 98 isolates showed the same profile with both methods. Among the 25 colistin resistant A. baumannii isolates identified by the BMD method, 23 presented the same profiles with the CBDE method, 1 isolate with a MIC of 4 μg/mL with the BMD method showed a MIC of >4 μg/mL with the CBDE method, and 1 heteroresistant isolate with a MIC of 8 μg/mL with the BMD method showed a MIC of 4 μg/mL with the CBDE method. None of the isolates showed the presence of *mcr* genes. Among the 25 colistin resistant A. baumannii isolates, 9 had a mutation in the *pmrA/B* and *lpxA/D* genes as the major mechanism of resistance, as previously characterized (3). The resistance mechanisms in the other 16 resistant isolates have not been investigated. The essential agreement (EA), categorical agreement (CA), sensitivity, and specificity for the CBDE method were 97.6% (*n* = 122), 98.4% (*n* = 123), 100%, and 98.40%, respectively. Major error was seen in 1.6% (*n* = 2) of the isolates, while no very major error was present. The EA and CA of the test are shown in Fig. 1, and the overall test results are summarized in Table 1.

**TABLE 1:** Summary of results comparing the BMD method with the CBDE method^*a*^

Study isolate	Total (n)	BMD		CBDE		EA (%)	CA (%)	ME (%)	VME (%)	Sensitivity (%)	Specificity (%)
		Col^S^	Col^R^	Col^S^	Col^R^						
Acinetobacter baumannii	125	100	25	98	27	97.6	98.4	1.6	0	100	98.40

a Col^S^: colistin susceptible; Col^R^: colistin resistant; EA: essential agreement; CA categorical agreement; ME: major error; VME: very major error. Both the methods, BMD and CBDE was done for 125 *A*. *baumannii* isolates and results were compared.

## DISCUSSION

There have been few studies on the topic of susceptibility testing in nonfermenters (NFs), despite several studies mentioning the issue. While one study clearly found higher error rates for NFs (major error: 33.33%; very major error: 12.5%), it considered testing only 9 isolates of A. baumannii (5). Similarly, another study also found error rates (major error: 3.3%; very major error: 5.6%) among 106 A. baumannii isolates (6). Conversely, another study interestingly found 100% EA and CA for 24 isolates of A. baumannii (7). In this regard, although the present study is the largest one, including 125 A. baumannii isolates, the number of colistin resistant isolates was only 25. This is one of the major limitations of the study. Nevertheless, isolates in the resistant category with both lower and higher MICs were included to address this limitation. All of the above studies reported concerns about the presence of plasmid-mediated colistin resistance through *mcr* genes and susceptibility testing by the CBDE method, though not in A. baumannii. In this regard, we suggest that for A. baumannii, in which the major colistin resistance mechanism is a mutational change (8), the CBDE method could be helpful, at least in resource-limited setups, versus not reporting susceptibility testing for colistin. However, the further evaluation of A. baumannii isolates with various MICs and different resistance mechanisms would better address the issue.

## MATERIALS AND METHODS

### Strains.

A total of 372 A. baumannii isolates from different clinical specimens, such as endotracheal aspirate, pus, blood, urine, and other body fluids, were collected from various inpatient and outpatient departments for inclusion in the study. The isolates had been primarily identified by standard biochemical methods (9) and a BD Phoenix M50 system (Becton, Dickinson and Company Diagnostics, India). The confirmation of these isolates as A. baumannii was done via a multiplex PCR assay targeting *recA* and the *ITS*-region gene (10). From those that were susceptible, a total of 100 isolates were randomly selected for susceptibility evaluation by the CBDE method, the results of which were compared with those obtained via the BMD method. In addition, 25 colistin resistant isolates were also included. Among these, 19 isolates had a MIC value of 16 μg/mL or above, 2 isolates each had MIC values of 8 μg/mL and 4 μg/mL, and 1 previously characterized heteroresistant isolate (by a population analysis profile) each had MIC values of 16 μg/mL and 8 μg/mL, respectively.

### Colistin susceptibility testing.

The MIC of colistin was tested by the broth microdilution method (2). The CBDE test was performed as described (2). Briefly, for each isolate, a set of 4 sterilized McCartney bottles was taken. The bottles were labeled with the required concentrations. 10 mL of cation-adjusted Mueller-Hinton broth media (HiMedia Laboratories Pvt Ltd., India) was evenly distributed into each bottle and kept for autoclaving. After the media cooled around 45°C, commercially available colistin disks (10 μg, Becton, Dickinson and Company Diagnostics, India) were added. For the proper elution of colistin into the medium, the bottles were gently vortexed and kept at room temperature for 30 min. 0, 1, 2, and 4 colistin disks were added to each bottle, generating final concentrations of 0 (growth control), 1 μg/mL, 2 μg/mL, and 4 μg/mL, respectively. For the inoculum preparation, 2 to 3 isolated pure colonies from overnight MacConkey agar plates were suspended into normal saline, and the turbidity was adjusted to a 0.5 McFarland standard. A 50 μL aliquot of the standardized suspension was added to each bottle. Then, the bottles were again vortexed and kept for overnight incubation at 35°C in ambient air. The MICs were visually read and interpreted according to the 2021 Clinical and Laboratory Standards Institute (CLSI) guidelines. P. aeruginosa ATCC 27853 and A. baumannii ATCC 19606 were used as standard controls. All of the colistin resistant isolates were tested for the presence of *mcr-1*, *mcr-2*, *mcr-3*, *mcr-4*, and *mcr-5* genes (3).

### Analysis of results.

A statistical analysis was performed for the comparison of the CBDE method against the gold standard BMD method (5). The sensitivity and specificity of the CBDE method were calculated using MedCalc statistical software version 19.6.3.0.
